# Will There Be Two Grades of Nurses?

**Published:** 1921-01-01

**Authors:** 


### THE MATRONS' AND SISTERS' DEPARTMENT.

### WILL THERE BE TWO GRADES OF NURSES?

Florence Nightingale was strongly of opinion that two grades of nurses were required. In building the Nursing Service she provided for the training of (*a*) women of education to organise and control, (*b*) the rank and file. She was probably influenced by military tradition, for it should never be forgotten that Army Nursing was the first branch of the Service to be systematically developed in the eye of the public. But theory and experience have gone hand in hand ever since in proclaiming the need for nursing officers of higher attainments than the main body of nurses. There is some danger at the present time of forgetting that this must always be the case.

The call for a sound minimum standard of training for the whole profession ought not to blind us to the fact that women of education in the present low state of education in this country, are not sufficiently numerous to give the public a Nursing Service of adequate size and variety. This is a fact no one at the present time is likely to dispute. We are compelled, therefore, to admit—and, indeed, we cordially welcome—women of a rather low educational standard to the training-schools, provided they be intelligent, sane, and trainable. This necessity for training women of unequal educational attainments has given rise to a problem which compels attention.

Is it just, or even strictly practical, to use the identical methods in training educated women which are found necessary in training probationers whose educational standard is low? Truth to tell, nearly all the difficulties of training arise out of this confusion. The incessant repetition, the constant levelling-down process found necessary with the one class is boring and wasteful with the other. At the same time, the stiffening process which is going on in some schools, in the endeavour to improve the general standard of nursing, discourages the women who stretch their minds with difficulty to face the very simplest form of test or examination.

If the nursing profession is to succeed in attracting educated probationers it must make some allowance for the indisputable fact that intelligent and cultivated women learn more quickly, assimilate more surely, and take less time to acquire the rudiments of their profession. How can this distinction be given its due weight save by making allowance for educated probationers to go forward on the road to efficiency?

The tyrant Procrustes chopped his victims to fit the size of the bed he prepared for them. Something of the same process can be observed in the training-schools compelled at the present time to prepare a mixed group of probationers to be nurses. It would seem there are two ways out of the difficulty, and both are already being tried.

The first is for certain training-schools to establish a preliminary standard of education and receive none who do not conform to it. The second is for training-schools with mixed groups to divide them into two standards under the sister-tutor, and give them lectures adapted to their several attainments. In either case, whether it be recognised or no, two distinct grades of nurses are being trained. To subject them to exactly similar examinations is the universal practice, and forms a curious anomaly in our training-system.

In the task of drawing all nurses within the net of registration it is of the utmost importance that the radical distinctions, not of class but of education and intelligence, should be kept in view. The nursing world awaits with a thrill of wonder and something like awe the regulations for a central examination of admission to the Register. There are no two opinions about the fact that it must be very easy, even if quite comprehensive, to fit the needs of the majority. But unless there be separate sets of papers for such as desire to take honours, and provision for qualifying in special subjects within the same period of time ordinarily devoted to the indispensable routine of general nursing, great discouragement will be given to the women of higher education we desire to see brought within our ranks. If they acquire practical details more quickly, assimilate lectures more thoroughly, are more useful in the wards, exercise a more wholesome influence on their subordinates, should not these qualities set them forward in time, as well as in degree, on the forward march? It is eminently discouraging to keep them three or four years grinding away like the rest at general nursing subjects, and then dismiss them to spend a year or two more in obtaining certificates indispensable to success in public work.

